# Initial Outcomes of an Online Continuing Education Series Focused on Post-treatment Cancer Survivorship Care

**DOI:** 10.1007/s13187-018-1453-2

**Published:** 2018-11-28

**Authors:** Allison Harvey, Yuqing Zhang, Serena Phillips, Rhea Suarez, Laura Dekle, Aubrey Villalobos, Mandi L. Pratt-Chapman

**Affiliations:** Institute for Patient-Centered Initiatives and Health Equity, The George Washington University Cancer Center, 2600 Virginia Avenue NW, Suite 300, Washington, DC 20037 USA

**Keywords:** Cancer survivorship, Comprehensive cancer control, Health care professional education, Evidence-based initiatives

## Abstract

There is a growing number of post-treatment cancer survivors in the USA. Cancer survivors can have a variety of care needs and health care professionals must be prepared to meet these needs. The American Cancer Society (ACS) and the George Washington University (GW) Cancer Center developed *The Cancer Survivorship E-Learning Series for Primary Care Providers* (E-Learning Series) to address the need for cancer survivorship training and education among health care professionals with a focus on primary care. The GW Cancer Center analyzed evaluation data from 1341 learners who voluntarily completed a module pre- and post-assessment between April 15, 2013, and December 31, 2017, to assess differences in self-rated confidence, on a five-point Likert scale, to meet learning objectives. Descriptive statistics characterize the sample and paired samples *t* tests were used to assess any statistically significant differences from pre to post (*p* < 0.05). Most learners were nurses (75.19%) and a majority of learners worked in oncology (74.68%) followed by primary care (11.60%). At pre-assessment, the module with the lowest mean self-confidence rating was 3.16 (*SD* = 0.81) and the highest was 3.60 (*SD* = 0.73). At post-assessment, module means in self-confidence rating ranged from 4.08 (*SD* = 0.46) to 4.26 (*SD* = 0.56). All differences were statistically significant (*p* < 0.0001). Results highlight gaps in confidence among health care professionals regarding cancer survivorship care and the need for continuing education. There is also a need for additional uptake of the E-Learning Series among primary care providers. Results suggest that the E-Learning Series is an effective educational tool that increases learners’ confidence in providing cancer survivorship care.

## Background

A growing evidence base indicates cancer survivors can face a host of long-term and late effects due to cancer and its treatment, which can affect survivors’ physical, emotional, spiritual, and social well-being. For example, cancer survivors are at an increased risk for developing second primary cancers [1], report higher levels of depression and anxiety compared to the general population [2], have difficulty adhering to medical care due to cancer-related financial hardship [3], and do not engage in recommend physical activity levels, which can improve overall quality of life and potentially decrease risk of recurrence [4]. Based on these and other needs, it is clear that the need for specialized health care services for cancer survivors does not end when primary treatment is finished. Due to the potential complexities, survivorship care requires a coordinated, multidisciplinary effort, involving oncology providers, primary care providers, other specialty providers, and public health professionals [5–7].

There are approximately 15.5 million cancer survivors living in the USA. It is projected the number of cancer survivors will grow to 20.3 million by 2026, an increase of 30% in 10 years [8]. More than a decade ago, cancer care stakeholders had the foresight that the US health care system would not be fully prepared to care for cancer survivors in the coming years. To help move cancer survivorship to the forefront, The National Academies of Sciences, Engineering, and Medicine (NASEM) Health and Medicine Division (formerly the Institute of Medicine) released its seminal 2005 report, *From Cancer Patient to Cancer Survivor: Lost in Transition*. The report identified several critical gaps, including the need for survivorship continuing education and training of diverse health care professionals. Per the NASEM report, education should address risks of treatments and cancer recurrence, management of psychosocial and late effects, and benefits of healthy lifestyle changes [9]. In addition to education and training, another key recommendation from the report was the creation and utilization of evidence-based cancer survivorship clinical care guidelines.

To help meet the call for education and development of guidelines, the American Cancer Society (ACS), the GW Cancer Center, and the Centers for Disease Control and Prevention (CDC) developed The National Cancer Survivorship Resource Center in 2010. Emerging from the five-year collaboration were key tools for health care professionals to fill identified gaps: *The Cancer Survivorship E-Learning Series for Primary Care Providers* (E-Learning Series); the *American Cancer Society Survivorship Clinical Care Guidelines* for prostate, colorectal, and head and neck cancers; and the *American Cancer Society/American Society of Clinical Oncology Breast Cancer Survivorship Clinical Care Guideline* [10–13]. These clinical guidelines provide evidence-based care recommendations for primary care providers and core content for four modules of the E-Learning Series.

This article provides an overview of the initial outcomes of the E-Learning Series, with a focus on reach as well as effectiveness of the series in increasing learner confidence in defined cancer survivorship learning objectives.

## Methods

### Training Development and Measures

The E-Learning Series is a no-cost, self-paced, 10-module online course and offers free continuing education credits/contact hours to physicians, nurses, physician assistants, and Certified Health Education Specialists (CHES®). Each module corresponds to 1 credit/contact hour. In order to receive credit, participants must successfully complete all activities of at least 1 module. Each module takes approximately 1 hour to complete and includes two 30-minute, evidence-based presentations from experts on the module topic. Each module also highlights the perspective of a cancer survivor and links to additional resources for providers. A learner can select which module(s) they want to take based on their interests and needs. When initially developed, the intended audience for the training was primary care providers; however, the training is open to anyone who wants to enroll.

The E-Learning Series launched on April 15, 2013 with three modules. The remaining modules were released on a rolling basis through March 31, 2016. The first six modules provide an overview of key components of cancer survivorship care, including management of comorbidities, psychosocial health, and tertiary prevention. The last four modules follow the same learning format but focus on the dissemination of the *American Cancer Society Survivorship Clinical Care Guidelines*.

The GW Cancer Center utilized the Kirkpatrick Evaluation Model to frame the evaluation of the E-Learning Series. This model is frequently used to evaluate trainings, including those for health care professionals [14, 15]. The GW Cancer Center focused evaluation on the first two levels of the model (Level 1: Learner satisfaction and Level 2: Learning outcomes) given the on-demand, online format of the learning platform.

### Study Population

This study did not meet the definition of human subjects research per guidance from the Institutional Review Board at the George Washington University. The rationale for this determination was that data were collected for the primary purpose of program evaluation and improvement for an online learning offering administered uniquely by GW and were not collected to encourage replication of the educational intervention outside of the local context. A total of 1656 unique learners voluntarily enrolled and participated in the E-Learning Series between April 15, 2013, and December 31, 2017, when data were exported for analysis. Learners who did not complete both a pre- and post-assessment for any given module or who indicated they practiced internationally were excluded from the sample. This evaluation includes learners practicing in the USA, tribes, and territories (*n* = 1341) who fully completed one or more modules’ pre- and post-assessments between launch and December 31, 2017.

### Online Training Promotion

The E-Learning Series has been marketed and disseminated nationally through the ACS, GW Cancer Center, and the CDC’s various communication channels, including social media and e-mail. In addition, the E-Learning Series has been posted on the National Commission for Health Education Credentialing, Inc. website and promoted at professional and regional conferences across the country.

### Data Collection

The GW Cancer Center administered the demographic survey and assessments and managed study data through SurveyMonkey® and our Learning Management System (LMS). Survey Monkey® was used from launch until December 2015, at which point the E-Learning Series pre/post evaluation data collection moved to the LMS.

At the beginning of the E-Learning Series, learners completed an optional demographic survey, which included questions on age, profession, field of practice, and place of practice. When the E-Learning Series moved to the LMS, additional questions were added such as self-identified gender, race, ethnicity, and practice setting (rural, suburban, urban, unsure or not applicable). Since questions were successively added or could be skipped, the sample size for demographic characteristics reported in Table 1 varies.

**Table 1:** Demographic characteristics of E-Learning Series participants

Demographic characteristics	Frequencies (%)
Age (*N* = 1328)	
21–29	133 (10.02)
30–39	284 (21.39)
40–49	344 (25.90)
50–59	388 (29.22)
60 or older	168 (12.65)
Prefer not to answer	11 (0.83)
Gender* (*N* = 1328)	
Female	1235 (93.00)
Male	86 (6.48)
Prefer not to answer	7 (0.53)
Race* (*N* = 701)	
Asian	52 (7.42)
American Indian or Alaska Native	1 (0.14)
Black	31 (4.42)
Native Hawaiian or Other Pacific Islander	1 (0.14)
Other	3 (0.43)
White	603 (86.02)
Multiracial	10 (1.43)
Ethnicity* (*N* = 741)	
Hispanic	34 (4.59)
Non-Hispanic	662(89.34)
Prefer not to answer	45 (6.07)
Region (Geographic) (*N* = 1262)	
Northeast	208 (16.48)
Midwest	403 (32.93)
South	434 (34.39)
West	217 (17.19)
Location (*N* = 741)	
Urban	329 (44.40)
Suburban	230 (31.04)
Rural	90 (12.15)
Unsure	30 (4.05)
Not applicable	62 (8.36)
Practice site* (*N* = 1328)	
Community health center	57 (4.29)
Hospital	453 (34.11)
Office practice	265 (19.95)
Outpatient center	372 (28.01)
Nonprofit organization	12 (0.90)
Government agency	21 (1.58)
Other practice site	123 (9.26)
Not applicable	25 (1.88)
Profession (*N* = 1322)	
Health care administrator	39 (2.95)
Physician	108 (8.17)
Nursing	994 (75.19)
Social worker	30 (2.27)
Patient navigator	17 (1.29)
Health educator	37 (2.80)
Other profession	97 (7.34)
Field of practice (*N* = 1319)	
Oncology	985 (74.68)
Primary care	153 (11.60)
Public health	21 (1.59)
Other field of practice	101 (7.66)
Not applicable	59 (4.47)

*Self-reported categories are as they appear on the demographic section of the Learning Management System

For each module, learners also completed a pre- and post-assessment. Each assessment had learners’ rate their agreement in their confidence to meet the module’s specific learning objectives. The number of learning objectives varied for each module, but averaged three to four objectives per module. The post-assessments also included questions on self-reported knowledge, strategies and skills gained, and intention to implement learnings in practice. Questions were measured on a five-point Likert scale (strongly disagree to strongly agree). An open-ended question was added later to the post-assessment, so learners could offer feedback or suggestions for improvement.

### Statistical Analysis

All statistical analyses were conducted using STATA®/IC 14.2. Response options from “profession” and “field of practice” were collapsed and made mutually exclusive prior to analysis. For “field of practice,” primary care was defined as family medicine, geriatrics, gynecology and internal medicine. For “profession,” nursing was defined as nurse, nurse practitioner, and nurse navigator. States were categorized and reported by US census region [16]. Both pre- and post-assessment ratings were averaged for each module. Post-assessment questions on self-reported knowledge, strategies and skills gained, and intention to implement information learned into practice were averaged across all modules then dichotomized by “agree/strongly agree” and “neutral/disagree/strongly disagree.” Descriptive statistics, including frequencies and percentages, were reported to quantify responses. Paired samples *t* tests were used to assess if the average difference in confidence from pre- to post-assessment was statistically significant for each individual learning objective, and when differences were averaged for each module. A *p* value of < 0.05 was considered statistically significant.

## Results

Demographic characteristics of the sample are presented in Table 1. The majority of learners identified as female (93.00%), White (86.02%), and non-Hispanic (89.34%). Most participants were between 40 and 59 years old. A majority of learners were nurses (75.19%) followed by physicians (8.17%). Learners worked mainly in oncology (74.68%) and some worked in primary care (11.60%). For practice location, most learners (44.40%) worked in an urban area, followed by suburban (31.04%) and then rural (12.15%). More than two thirds of learners (67.32%) were from the South and Midwest regions of the USA.

Sample size for each module, module topic, and launch date are presented in Table 2. Participation was higher for Modules 1 through 4 and tapered for remaining modules with less than 500 learners completing each module. Learning objectives for each module are presented in Table 3.

**Table 2:** E-Learning Series modules, launch date, and number of learners

Module #	Module title/topic	Launch date	# learners
Module 1	The Current State of Survivorship Care and the Role of Primary Care Providers	April 2013	1041
Module 2	Late Effects of Cancer and its Treatments: Managing Comorbidities and Coordinating with Specialty Providers	April 2013	715
Module 3	Late Effects of Cancer and its Treatment: Meeting the Psychosocial Health Care Needs of Survivors	April 2013	751
Module 4	The Importance of Prevention in Cancer Survivorship: Empowering Survivors to Live Well	December 2013	528
Module 5	A Team Approach: Survivorship Care Coordination	December 2013	315
Module 6	Cancer Recovery and Rehabilitation	April 2014	440
Module 7	Spotlight on Prostate Cancer Survivorship: Clinical Follow-Up Care Guideline for Primary Care Providers	July 2014	368
Module 8	Spotlight on Colorectal Cancer Survivorship: Clinical Follow-Up Care Guideline for Primary Care Providers	September 2015	304
Module 9	Spotlight on Breast Cancer Survivorship: Clinical Follow-Up Care Guideline for Primary Care Providers	December 2015	294
Module 10	Spotlight on Head and Neck Cancer Survivorship: Clinical Follow-Up Care Guideline for Primary Care Providers	March 2016	225

**Table 3:** E-Learning Series learning objectives by module

Module #	Learning objectives
1	1. I am confident in my knowledge of models of cancer survivorship follow-up care.
	2. I am confident in my ability to describe national efforts related to survivorship care.
	3. I am confident in my understanding of a primary care provider's (PCP’s) role in providing care to cancer survivors.
2	1. I am confident in my ability to describe common late effects after treatment with chemotherapy, radiation therapy, hormone therapy, and surgery.
	2. I am confident in my ability to describe how cancer treatment late effects may interact with other non-cancer comorbidities.
	3. I am confident in my ability to implement a coordinated plan of care/consult with specialty providers to manage medical late effects of cancer when appropriate.
3	1. I am confident in my ability to identify types of psychosocial issues and how they vary based on time since treatment completion.
	2. I am confident in my ability to describe risk factors for psychosocial consequences of cancer and its treatment.
	3. I am confident in my ability to describe how to screen for distress and the PCP’s role in follow-up psychosocial care.
	4. I am confident in my ability to provide appropriate psychosocial care to post-treatment cancer survivors.
4	1. I am confident in my ability to explain the PCP role in providing survivorship care focused on prevention, wellness, and evidence-based guidelines for screening.
	2. I am confident in my ability to provide guideline-supported recommendations for secondary prevention to cancer survivors regarding sunscreen, diet, obesity, exercise, alcohol, and tobacco.
	3. I am confident in my ability to explain the importance of prevention and wellness in cancer survivorship care.
5	1. I am confident in my ability to describe the roles of oncologists and primary care providers in the shared-care model.
	2. I am confident in my ability to explain the importance of the survivorship care plan as a communication tool to coordinate care between the oncologist and primary care provider.
	3. I am confident in my ability to describe the role of the primary care provider in providing follow-up care to cancer survivors in the primary care setting.
	4. I am confident in my ability to describe coordination of care between oncologists and primary care providers in transitioning a patient from oncology to primary care.
6	1. I am confident in my ability to understand the role and importance of rehabilitation post-treatment.
	2. I am confident in my ability to understand the role and importance of spirituality during recovery post-treatment.
	3. I am confident in my ability to identify interventions to assist in physical, emotional, and spiritual recovery of cancer survivors.
7	1. I am confident in my ability to describe the potential long-term and late effects of prostate cancer and its treatment.
	2. I am confident in my ability to describe how to care for prostate cancer survivors as outlined in the new American Cancer Society Prostate Cancer Survivorship Care Guideline.
	3. I am confident in my ability to demonstrate understanding of a PCP’s role in providing follow-up care to prostate cancer survivors.
	4. I am confident in my ability to appropriately utilize current clinical guidelines when providing care to prostate cancer survivors.
8	1. I am confident in my ability to describe potential late and long-term effects of disease or treatments for colorectal cancer survivors.
	2. I am confident in my ability to describe how to care for colorectal cancer survivors as outlined in the new American Cancer Society Colorectal Cancer Survivorship Care Guideline.
	3. I am confident in my ability to explain a PCP’s role in providing clinical follow-up care to colorectal cancer survivors.
9	1. I am confident in my ability to describe potential long-term and late effects of breast cancer and its treatments.
	2. I am confident in my ability to describe how to care for breast cancer survivors as outlined in the American Cancer Society/American Society of Clinical Oncology Breast Cancer Survivorship Guideline.
	3. I am confident in my ability to explain a Primary Care Clinician’s role in providing clinical follow-up care to breast cancer survivors.
10	1. I am confident in my ability to describe potential long-term and late effects of head and neck cancer and its treatment.
	2. I am confident in my ability to describe how to care for head and neck cancer survivors as outlined in the American Cancer Society Head and Neck Cancer Survivorship Care Guideline.
	3. I am confident in my ability to describe a primary care clinician’s role in providing clinical follow-up care to head and neck cancer survivors.

*PCP* primary care provider

Reviewing learners’ self-rated confidence from pre- to post-assessment (Table 4), there was an increase in mean self-confidence rating for aggregated learning objectives from pre to post for each module, which ranged from 0.66 (*SD* = 0.67) to 0.92 (*SD* = 0.77). Change in mean confidence rating was statistically significant (*p* < 0.0001) for all modules, and the pre-post differences for each individual learning objective were statistically significant (*p* < 0.0001) (individual objectives’ change scores are not presented in the table). For pre-assessment, the average aggregate confidence rating for learners per module ranged from 3.16 (*SD* = 0.81) to 3.60 (*SD* = 0.73). Self-reported confidence in the ability to meet learning objectives was lowest in Module 10 (3.16) and Module 1 (3.20). In the post-assessment, the average aggregate confidence rating for learners per module ranged from 4.08 (*SD* = 0.46) to 4.26 (*SD* = 0.56). Also at post-assessment, on average a majority of learners agreed or strongly agreed their knowledge was enhanced (91.59%), they gained new strategies/skills/information they could apply to practice (83.41%), and planned to implement these gains into practice (75.38%).

**Table 4:** E-Learning Series average pre- and post-assessment means and change in learners’ self-confidence rating

Module #	Average pre-assessment mean (SD)	Average post-assessment mean (SD)	Average change in rating from pre/post (SD)	*P* value (for pre/post change)
1	3.20 (0.83)	4.10 (0.56)	0.91 (0.79)	< 0.0001*
2	3.39 (0.82)	4.14 (0.57)	0.75 (0.72)	< 0.0001*
3	3.47 (0.75)	4.18 (0.55)	0.71 (0.68)	< 0.0001*
4	3.60 (0.73)	4.26 (0.56)	0.66 (0.67)	< 0.0001*
5	3.54 (0.74)	4.25 (0.53)	0.71 (0.66)	< 0.0001*
6	3.54 (0.75)	4.22 (0.55)	0.68 (0.70)	< 0.0001*
7	3.24 (0.81)	4.12 (0.54)	0.88 (0.77)	< 0.0001*
8	3.32 (0.77)	4.10 (0.52)	0.79 (0.72)	< 0.0001*
9	3.40 (0.81)	4.13 (0.55)	0.73 (0.72)	< 0.0001*
10	3.16 (0.81)	4.08 (0.46)	0.92 (0.77)	< 0.0001*

## Discussion

Initial evaluation outcomes support the need for ongoing continuing education in cancer survivorship for all health care professionals as identified in the NASEM report more than a decade ago. When the E-Learning Series was developed, the intended audience was primary care providers. However, based on participation across fields, results suggest that diverse health care professionals are in need of and are seeking information about cancer survivorship.

Results also highlight baseline gaps in confidence among health care professionals regarding cancer survivorship care and suggest the E-Learning Series is effective in reducing these deficits. For example, at the beginning of each module, on average, learners rated their confidence to meet learning objectives as “neutral.” Upon module completion, learners’ confidence on average increased by almost one point, which indicates that most learners agreed they were confident in their ability to meet learning objectives. The learning objective with the greatest increase from pre to post (1.01 points) was “I am confident in my ability to describe national efforts related to survivorship care” (Module 1), suggesting that most learners were unaware of larger national efforts. Integration of individual-level provider behavior with national benchmarks is critical to advance broader health systems change, particularly in the area of care coordination across providers. Observed gains from pre- to post-assessment demonstrate that the E-Learning Series is an effective educational tool to increase learner confidence in addressing physical and psychosocial aspects of cancer survivorship care.

The E-Learning Series reached learners across the USA, yet results indicate the need for additional dissemination and uptake of this educational tool particularly among primary care providers. The 1341 learners who have taken this online training cannot meet the needs of a population of 15.5 million cancer survivors. Modules that focus on follow-up clinical care guidelines for primary care providers (Modules 7–10) have lower participation compared to earlier modules. This could be due to the fact that these modules were released later and have had less time to accrue learners and also due to their disease-specific and clinical nature. Dissemination and uptake of clinical guidelines are critical to move evidence into practice. To support additional dissemination and uptake of the E-Learning Series by primary care providers, the GW Cancer Center recently developed an evidence-based communication toolkit [17] to help health care organizations and primary care professional membership associations promote this learning opportunity. The toolkit includes sample social media messages, e-mails, and blog posts to increase interest in the E-Learning Series. Messages highlight the self-paced nature of the training, the critical role primary care providers play in post-treatment survivorship care, and the need for providers to participate in continuing education to improve cancer survivors’ care coordination and health outcomes [18]. The toolkit has been downloaded more than 3500 times since its release in July 2018.

Another possible barrier to uptake is the online delivery of the program. Diverse learners have different preferences for learning formats. Feedback from learners includes technological challenges, such as audio or visual malfunctions, which may have caused some learners to not complete a module. The GW Cancer Center updates modules on a rolling basis as issues are reported in order to improve the user experience and to ensure evidence presented is current. The GW Cancer Center anticipates updating Module 5 in the coming year, since new evidence regarding models of care and the use of survivorship care plans has emerged since the module was released in 2013. Additional methods for dissemination, contingent on resources, will be explored to increase access and uptake.

Limitations of the present analysis include lack of a comparison group, a convenience sample of learners, and the self-reported nature of the data. Future directions for research should include retention of learner confidence longitudinally, as well as behavioral intention, application to practice, such as coordination of care, uptake and impact of recommended guidelines, and patient-reported outcomes.

## Conclusions

There is a need for continued education and training of health care professionals to equip them with the knowledge and skills to care for a growing population of post-treatment cancer survivors. The E-Learning Series is an effective educational tool to increase health care providers’ confidence in cancer survivorship care. Additional dissemination of this educational tool is warranted to build capacity of a health care professional workforce responsive to the diverse needs of the growing population of cancer survivors.
